# Supplementary material for: Complexome profiling of the Chlamydomonas psb28 mutant reveals TEF5 as an early PSII assembly factor
Source: Plant Cell. 2025 Mar 17;37(6):koaf055. doi: 10.1093/plcell/koaf055 (PMC12164590; doi:10.1093/plcell/koaf055)
Supplement: koaf055_Supplementary_Data [file koaf055_supplementary_data.zip › psb28_interactive.zip!/plots/intensity/qualityPlots/peptides/APX4_Cre05.g233900.t1.2.html]

### Cre05.g233900.t1.2

Trivial name: APX4  
SeqCoverage [median]: 34.30  
Mass: 36.49 kDa  
  
 

| Sequence | MissedCleavage | Mass [Da] | wtIntensity | muIntensity | Score |
| --- | --- | --- | --- | --- | --- |
| AFAATLGAK | 0 | 848.48 | 14288.21 | 17590.14 | 113.41 |
| AQIASGGEVIAGPAFSAAWPVR | 0 | 2154.12 | 2029.26 | 0.00 | 113.40 |
| LGRVDSPAAAAVK | 1 | 1253.71 | 12475.61 | 7749.39 | 122.39 |
| LGRVDSPAAAAVKVPTYDSPVAEIK | 2 | 2553.38 | 3350.22 | 3584.61 | 96.54 |
| LVLNDAADWDPVTK | 0 | 1555.79 | 9512.27 | 9628.01 | 176.97 |
| TGGVNGSIVTPEALARPENAGLEVVVAK | 1 | 2747.48 | 810.90 | 0.00 | 39.89 |
| VDSPAAAAVK | 0 | 927.50 | 21459.53 | 20074.06 | 136.80 |
| VDSPAAAAVKVPTYDSPVAEIK | 1 | 2227.17 | 8376.20 | 10455.87 | 123.99 |
| VPTYDSPVAEIK | 0 | 1317.68 | 23383.88 | 33871.74 | 218.11 |
| VTTQSGWSATK | 0 | 1164.58 | 11140.86 | 1908.75 | 160.77 |
| WVSEDPATFK | 0 | 1178.56 | 10450.86 | 5501.40 | 128.38 |
|  |  |  | 117277.8 | 110364.0 |  |
